# The Influence of SARS-CoV-2 Pandemic on the Diagnosis of Celiac Disease and Clinical Practice in Pediatric Gastroenterology

**DOI:** 10.3390/nu15030559

**Published:** 2023-01-21

**Authors:** Marco Crocco, Angela Calvi, Francesca Canzoneri, Federica Malerba, Noemi Zampatti, Andrea Chiaro, Serena Arrigo, Paolo Gandullia, Stefania Proietti, Stefano Bonassi

**Affiliations:** 1 Pediatric Gastroenterology and Endoscopy Unit, IRCCS Istituto Giannina Gaslini, 16147 Genoa, Italy; 2 Department of Neuroscience, Rehabilitation, Ophthalmology, Genetics, Child and Maternal Health, University of Genova, 16132 Genova, Italy; 3 Department of Human Sciences and Quality of Life Promotion, San Raffaele University, 00166 Rome, Italy; 4 Unit of Clinical and Molecular Epidemiology, IRCCS San Raffaele Pisana, 00166 Rome, Italy

**Keywords:** celiac disease, COVID-19, SARS-CoV-2, pandemic, pediatric gastroenterology, lockdown, gluten free diet, coeliac, gluten disorder

## Abstract

Celiac disease (CD) has a high prevalence but remains largely underdiagnosed. Although extensive studies have confirmed that children with CD do not have an increased risk of severe COVID-19, public health regulations associated with the SARS-CoV-2 pandemic may have exacerbated this problem. The aim of this study was to assess the effect of SARS-CoV-2 on the number of new-onset CD cases. Additionally, the role of SARS-CoV-2 in autoimmune diseases and its influence on clinical practice in pediatric gastroenterology were briefly reviewed. We described the data from the hospital electronic registry of new-onset CD, during the COVID-19 pandemic and 2 years before. A total of 423 children were diagnosed with CD between March 2018 and February 2022: 228 in the 2-year pre-COVID-19 period and 195 during the pandemic. The number of patients during the COVID-19 pandemic was 14.5% lower than in the previous years. The quarterly comparison of CD diagnoses showed a reduction in all quarters. A reduction in diagnoses during the lockdown and in the following months was evident and not compensated thereafter. This is the first study to evaluate the impact of SARS-CoV-2 on the diagnosis of CD in children. Further studies are necessary to improve the system of biopsy-sparing diagnosis and to evaluate the effect of the diagnostic delay. Special attention should be given to the implementation of telemedicine services.

## 1. Introduction

Celiac disease is a chronic immune-mediated disease triggered by the ingestion of gluten in genetically susceptible individuals. Clinical symptoms are heterogeneous due to gastrointestinal and extraintestinal disorders. The etiopathogenesis of CD is multifactorial, involving both environmental and genetic factors. Environmental exposure during childhood plays an important role in the development of CD and other autoimmune disorders. A virus infection may lead to an autoimmune disorder via various pathogenic mechanisms [1]. Viruses can disrupt virus–host intestinal immune homeostasis and initiate loss of oral tolerance and TH1 immunity to gluten [2]. Several viruses have been investigated as triggering mechanisms including adenovirus, enteroviruses, hepatitis C virus, reovirus, and rotavirus [3].

Despite epidemiological evidence of associations between SARS-CoV-2 and an increased incidence of autoimmune disorders in children such as diabetes type 1 [4] and multisystem inflammatory syndrome [5], little is known about the effect of the COVID-19 pandemic on the incidence of CD.

Children are less commonly affected by COVID-19 than adults and usually exhibit mild symptoms [6]. This variation in clinical presentation might be explained by the different innate and adaptive immune responses, the presence of pre-existing cross-reactive T-cell response to seasonal coronaviruses (not SARS-CoV-2), and lower rates of comorbidities associated with severe COVID-19 in childhood [7].

There is general agreement that the consequences of SARS-CoV-2 infection in celiacs are more related to quality of life than to organ damage [8]. Preliminary data from the International Coronavirus and Celiac Disease Reporting Database showed that CD serology and adherence to a gluten-free diet (GFD) were not associated with severe outcomes [9]. These results were confirmed by subsequent studies, which did not find a correlation between COVID-19 and CD, reporting a similar clinical course in celiac and nonceliac patients, especially in those compliant with the GFD [10,11].

Due to the SARS-CoV-2 pandemic and the consequent limitations of endoscopic procedures and general access to hospitals, several practical recommendations have been proposed for the diagnosis and follow-up of CD in this period. A biopsy-sparing approach was proposed for the diagnosis of CD in adults [12,13]; for children, a reduction to ≥5–7.5 times of the upper limit normal of the antitransglutaminase IgA threshold was considered [14,15,16]. Due to the exponential growth of the incidence of SARS-CoV-2 infection and the consequent risk of the collapse of the national health system, the Italian government implemented containment policies aimed at mitigating the spread of the disease. These policies included a general lockdown issued on 9 March 2020. This resolution remained in place until 3 May 2020 and implied that all people in the country had to remain confined to their homes. These containment policies and fear of SARS-CoV-2 infection may have affected the process of CD diagnosis in the last two years, especially during the period of lockdown. 

The aim of this study was to assess the effect of SARS-CoV-2 on the number of new-onset CD cases in a tertiary pediatric center. Additionally, we discussed the influence of the COVID-19 pandemic on pediatric clinical practice in gastroenterology.

## 2. Materials and Methods

The study was conducted as a retrospective evaluation of all new-onset of CD diagnosed by the Department of Gastroenterology of the Giannina Gaslini Children Hospital, Genoa, during the COVID-19 pandemic (COVID group) and compared with newly diagnosed patients in the prepandemic 2-year period (control group). The study was conducted in accordance with the Declaration of Helsinki and approved by the Ethics Committee of Liguria (4019/22).

We extracted the data of all new CD diagnoses in the two groups from the hospital’s electronic registry used for mandatory communication to the regional health system. Only children younger than 18 years of age with a first confirmed diagnosis between 1 March 2018 and 28 February 2022 were included in the analysis. All diagnoses were confirmed by histological examination of duodenal biopsies or according to the 2020 clinical criteria of the European Society for Pediatric Gastroenterology Hepatology and Nutrition (ESPGHAN) [17]. All new diagnoses were re-evaluated by the same pediatric gastroenterologist (AC). Patients with potential or suspected CD in which the diagnostic protocol was still ongoing were excluded. 

The mean number per trimester was calculated for newly diagnosed patients in the prepandemic period (1 March 2018, to 29 February 2020) and compared with the number of patients diagnosed during the pandemic period (1 March 2020 to 28 February 2022). Descriptive statistics are presented as frequencies, percentages, means, and standard deviations (SDs). The chi-square test was used to compare the frequency of newly diagnosed patients during the prepandemic and pandemic periods. To evaluate the presence of autocorrelation in the time distribution of diagnoses the Ljung–Box test for time series was applied. This test examines, for each lag (period of time = month), the autocorrelation by asymptotic approximation of the chi-squared. A *p* value < 0.05 (nonstationary signal) is consistent with a nonindependent distribution of residuals, which then exhibit serial correlation. Statistical analysis was performed using SPSS statistical software (version 28.0, IBM Corp., Armonk, NY, USA).

## 3. Results

**Figure 1:**
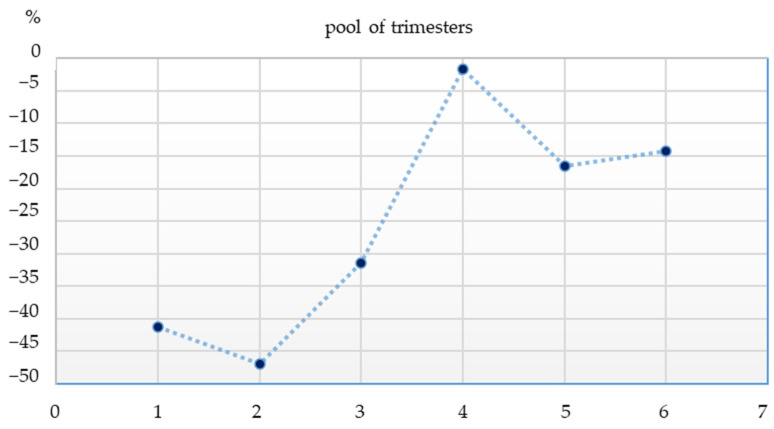
Quarterly comparison of CD diagnoses in the pre-COVID-19 period (pool of trimesters from March 2018 to February 2020) vs. the corresponding trimesters of the COVID-19 pandemic period (March 2020–February 2022).

A total of 423 children were diagnosed with CD between March 2018 and February 2022 at our pediatric regional center: 228 in the 2-year pre-COVID-19, and 195 during the COVID-19 pandemic period. The total number of patients during the two years of the COVID-19 pandemic was 14.5% lower than in the two previous years. The quarterly comparison of CD diagnoses in the pre-COVID-19 period (pool of trimesters from March 2018 to February 2020) vs. the corresponding trimesters of the COVID-19 pandemic period (March 2020–February 2022) showed a nonstatistically significant reduction in diagnoses in all trimesters, more evident in the first part of 2020 (−41.7%, −46.9%, and −31.4%) and, to a lesser extent, in 2021 (Figure 1). 

**Figure 2:**
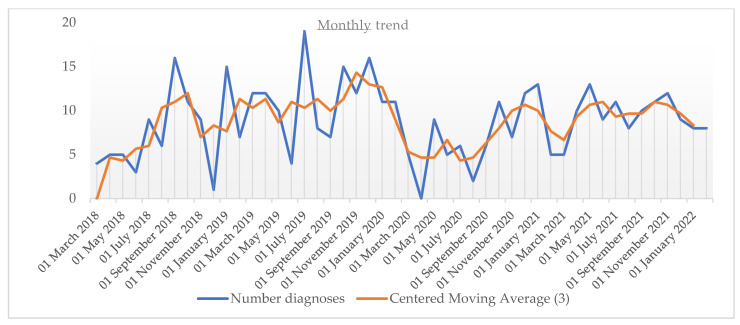
Monthly diagnosis rates and relative moving average (order 3) between March 2018 and February 2022.

A more detailed description of monthly CD diagnoses showed a reduction in the rate during the period of the first lockdown and in the following months, until the mini lockdown in October 2020, which roughly correspond to the first and second waves of the pandemic. In particular, no CD diagnoses were reported between late March and late May 2020. This period corresponded to the first legal lockdown (a stay-at-home order) with a shutdown of all activities. A stable, lower number of diagnoses was noted thereafter. No evidence of recovery of missing diagnoses was detected (Figure 2). The quarterly diagnosis rates for the same time frames are shown in Supplementary Figure S1. 

**Figure 3:**
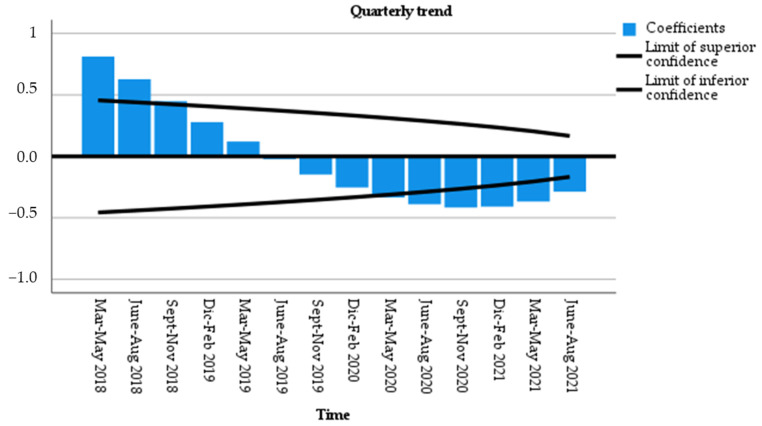
Autocorrelation function (ACF) and partial autocorrelation plots: seasonal differencing of quarters from March 2018 to August 2021.

The time series analysis of data, i.e., the ability of each trimester to predict the number of new diagnoses in the following trimester, revealed a clear tendency to reduce the degree of autocorrelation between trimesters in the period immediately before the first lockdown (Ljung–Box test borderline significant). The approximation to zero of the autocorrelation factor (ACF) at the end of the observation revealed the likely end of the perturbation, confirming the lack of recovery for missing diagnoses (Figure 3).

## 4. Discussion

The discussion of the extent to which the SARS-CoV-2 pandemic has affected the ability of referent clinical centers to diagnose new cases of CD and, in general, of the changes in the clinical practice for pediatric gastroenterologists is divided into two sections. 

### 4.1. The Influence of SARS-CoV-2 Pandemic on Pediatric Gastroenterology Clinical Practice

Recent studies demonstrated that patients with inflammatory bowel disease (IBD) have no risk or are at low risk of developing COVID-19 [18]. Similarly, patients with CD do not seem to have an increased risk of COVID-19 compared with the general population, and, if infected, their disease course is similar to that of the general population [11]. However, the COVID-19 pandemic has had a major effect on pediatric gastroenterology practice [19], and a potentially life-threatening delay in the diagnosis of celiac disease was also reported in a young child [20]. 

According to existing data, during the pandemic period, the numbers of both clinic visits for gastrointestinal disorders and new hospital admissions through the emergency departments have drastically decreased [21,22]. In addition, due to diagnostic delays, an increased rate of complicated appendicitis, pyloric stenosis, sepsis, and cancer has been reported [23,24,25]. Despite medical services provided by national and regional health systems being guaranteed during the pandemic period (at least for clinical emergencies), during lockdown periods, the access to hospital and clinics was limited to visits that could not be delayed. On 20 March 2020, the Italian Federation of Societies of Diseases of the Digestive System suspended ordinary outpatient activities, limiting access to nondeferrable emergency cases and cancer patients [26]. In April 2020, the European Society of Gastrointestinal Endoscopy and the European Society of Gastroenterology and Endoscopy Nurses and Associates published a position paper stating that gastrointestinal endoscopy units should strongly consider temporarily postponing elective, nonurgent endoscopy procedures [15]. Up to 90% of the visits in March and April 2020 were postponed, and many were switched to telemedicine [27]. 

Telemedicine has shown great potential for coping with the difficulty of providing medical assistance in this period, although it cannot fully replace clinical practice and may create health inequalities based on income and access to e-technology.

### 4.2. The Influence of SARS-CoV-2 Pandemic on the Frequency of New Diagnoses of CD in a Pediatric Gastroenterology Tertiary Center

In the last decades, the number of patients diagnosed with CD has increased worldwide due to improved diagnostic protocols, as well as the expansion of the knowledge, recognition, and testing for the disease [28,29]. The number of CD patients diagnosed in Italy increased from 66,398 in 2007 to 233,147 in the 2020. In the same period, the prevalence of diagnosis progressively increased from 0.11% to 0.39%, with a downtrend in new diagnoses since 2017. Nevertheless, these figures are still far from the expected prevalence of 1–2% resulting from cohort studies [30]. Several scientific publications appeared on CD during the pandemic, although the influence of the infection on the incidence of CD is unclear, and data are still lacking. Here, we describe the effect of the COVID-19 pandemic on the rate of new CD diagnoses in pediatric patients. To the best of our knowledge, no study has yet been published evaluating this topic. 

There are many possible causes of CD underdiagnosis in children described in our study. Our department serves as a regional referral center for CD for all Ligurian provinces, and there were fewer referrals from other secondary centers and fewer hospital visits during the pandemic. In Liguria, the prevalence of diagnosis progressively increased from 0.33% (+247 cases in 2017) to 0.34% (+116 cases in 2018)–0.36 (+233 cases in 2019) to 0.38% (+210 cases 2020). However, in Italy, the uptrend in the number of CD diagnoses over the years has been fluctuating in recent years: +4.1% (+8134 cases in 2017), +3.7% (+7678 cases in 2018), +5.2% (+11,179 cases in 2019), and 3.4% (+7729 cases in 2020). Therefore, the result of underdiagnosis in the COVID-19 pandemic period could have also been related to fluctuations in the number of diagnoses present even before COVID-19.

During the lockdown periods in Italy, corresponding to the waves of SARS-CoV-2 infections, a drastic reduction in endoscopic procedures performed for suspected CD (−55.1%) and other gastrointestinal disorders were reported. The same study also observed a significant reduction in the total number of endoscopies performed for common symptoms of CD, such as recurrent abdominal pain (−54.7%), anemia (−39.4%), weight loss (−38.1%), and dyspepsia (−49.8%) [31]. A possible CD diagnostic delay can certainly be attributed to decreased access to healthcare, although the fear of COVID-19, as reported for other diseases [23,32], may have contributed. The hospital routine endoscopic activities for the confirmation of CD diagnosis in patients who were not compliant with the serologic parameters of the ESPGHAN guideline restarted in late September 2020, contributing to the partial recovery of diagnosis in the last part of that year. 

On the other hand, an absolute reduction in the number of newly diagnosed patients may have also been related to new factors that are currently unknown. Growing evidence supports the role of individual and environmental exposome in the incidence of autoimmune diseases [33,34]. Several birth cohort studies have reported the association of gastrointestinal infections with transglutaminase antibody seroconversion and an increased risk of CD later in life [35,36]. Virus infections are a possible factor responsible for the seasonality in the risk of CD, and vaccination against viruses such as the rotavirus are considered to have a protective effect [37]. Importantly, these mechanisms are often promoted by asymptomatic and self-limiting gastrointestinal virus infections during childhood [2,38]. During the COVID-19 period, many hygienic measures were implemented, including the use of face masks, social distancing, and travel restrictions. As a result, the transmission of other viruses was also largely reduced; therefore, it may have led to a decrease in the prevalence of some autoimmune diseases triggered by infections. Despite the fact that exposure to SARS-CoV-2 may initiate gastrointestinal inflammation supports the hypothesis that SARS-CoV-2 may trigger autoimmune mechanisms in genetically predisposed subjects [39,40,41], a protective effect of SARS-CoV-2 infection on the occurrence of CD cannot be excluded a priori. Some human leukocyte antigens are implicated in the autoimmune reactivity triggered by the viruses [42,43,44], other than the resulting protective effect against some viral diseases such as HIV and dengue [45,46]. Furthermore, during the lockdown, the reduction in environmental pollution and the changes in diet and lifestyles may have positively influenced the complex homeostasis between the microbiome and the immune system and, therefore, may have had a protective effect on the onset of autoimmune diseases, including CD. Some dietary and lifestyle habits, such as fresh food consumption and routine physical activity, are positively correlated with time spent at home [47,48].

Cellular and epidemiological studies are needed to understand the possible involvement of the SARS-CoV-2 in the etiopathogenesis of CD.

Among the limitations of the study, it should be considered that our data were from administrative records and, therefore, are more prone to error, especially for the exact timing of diagnosis. However, the delay between diagnosis and administrative communication is generally no longer than a few weeks, limiting the effect of this discrepancy. Our results on the pediatric population are in line with national data published by the Minister of Health on the general population. However, this is a retrospective monocentric study; therefore, its application cannot be generalized, especially for adults or to the national population. In fact, many adult gastroenterology units in Italy were converted to COVID-19 units for a long time, healthcare staff did not have sufficient personal protective equipment, and operating rooms were reserved to support intensive care units. 

It would be important to compare our data with those of other countries where, during the pandemic emergency, even in adulthood, diagnosis was performed without biopsy in a large number of cases. To the best of our knowledge, no data are available from other centers performing CD diagnosis with a biopsy-sparing protocol in adults, as recommended by the interim indication of the British Society of Gastroenterology (BSG) [12], or in symptomatic children using the threshold of ×7.5 times the value of the norm of antitransglutaminase IgA, as recommended by the interim indications of the Italian Association of Pediatric Gastroenterology, Nutrition and Hepatology [49]. 

As recommended by the Italian Ministry of Health, almost all centers have implemented the use of telemedicine in the diagnosis and follow-up of CD. The SARS-CoV-2 pandemic was the first occasion in Italy in which doctors and patients carried out permanent remote consultations. This innovation has proved to be very useful, documented by the fact that the large majority of patients who have relied on remote counseling services for disease monitoring reported being satisfied. This suggests that teleconsultation may become standard in CD healthcare. 

## 5. Conclusions

While extensive studies have confirmed that children with CD do not have an increased risk of severe COVID-19, much less has been reported on the diagnostic delay during the COVID-19 pandemic, which has contributed to a decrease in the CD diagnosis rate. The prevalence of undiagnosed CD is still high; therefore, it will be necessary to reduce the gap caused by the pandemic to prevent the number of undiagnosed people from increasing even farther. We reported, for the first time, data on the diagnostic delay in CD children during the pandemic period. 

The careful evaluation of the trends in pediatric gastrointestinal diseases during the COVID-19 pandemic, including the study of outcomes and the consequences of the diagnostic delay, should be a priority for both physicians and administrators. Multicenter retrospective and prospective studies are still necessary to quantify the gap, to improve the system of biopsy-sparing diagnosis, to facilitate access to the protocols, and to evaluate the possible damage caused by diagnostic delay in the coming years and whether there will be a rebound in diagnoses. Interventions may be required to fill the diagnostic gap of CD.

Although the pandemic has helped to demonstrate the usefulness and feasibility of telemedicine, the permanent and widespread adoption of telemedicine services is complex and must be tailored according to the clinical case.

## Data Availability

The data presented in this study are available on request from the corresponding author.

## Supplementary Materials

The following supporting information can be downloaded at: https://www.mdpi.com/article/10.3390/nu15030559/s1, Figure S1: Quarterly diagnosis rates between March 2018 and February 2022.
