# Supplementary figures and images for: The Influence of SARS-CoV-2 Pandemic on the Diagnosis of Celiac Disease and Clinical Practice in Pediatric Gastroenterology
Source: Nutrients. 2023 Jan 21;15(3):559. doi: 10.3390/nu15030559 (PMC9920531; doi:10.3390/nu15030559)

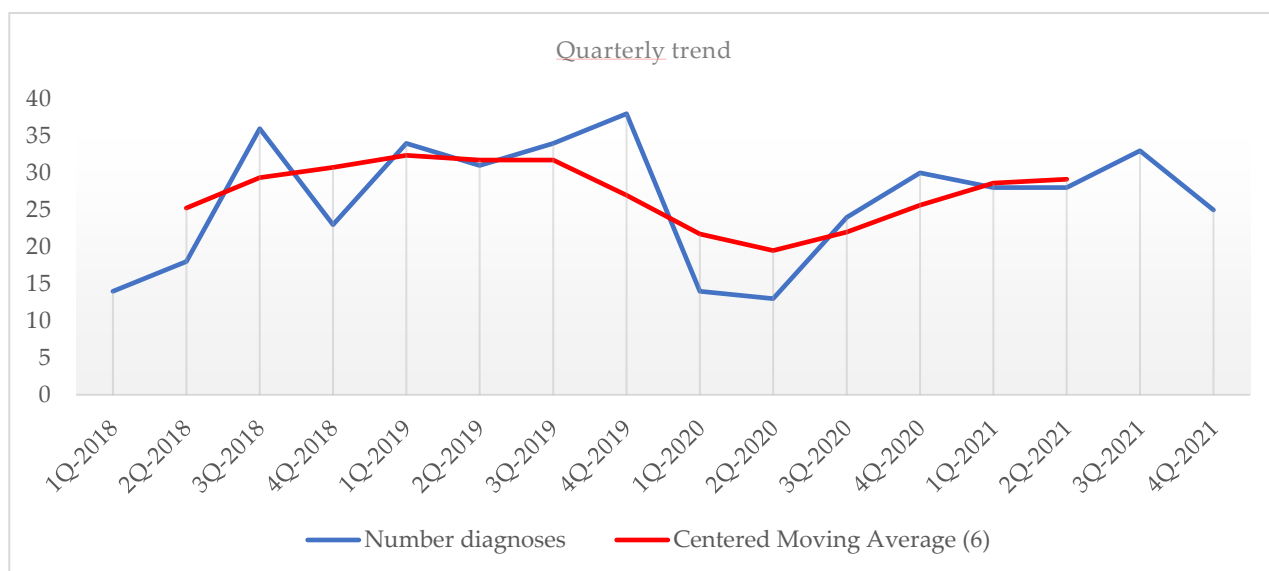

**Supplementary Figure S1.** Quarterly diagnosis rates between March 2018 and February 2022.

Supplement: Supplementary file 1 [file nutrients-15-00559-s001.zip › nutrients-2095498-supplementary.pdf]
